# Compatibility of hypokalaemia caused by low-dose prednisolone plus abiraterone acetate therapy for metastatic castration-resistant prostate cancer

**DOI:** 10.1186/s40780-024-00391-5

**Published:** 2024-11-11

**Authors:** Shota Torii, Aya Torii-Goto, Tamaki Tanizawa, Takashi Sakakibara, Ryosuke Oguri, Haruka Nagase, Yuri Nakao, Tomoyuki Hirashita, Kuniaki Tanaka, Norio Takimoto, Takahiro Hayashi

**Affiliations:** 1 Department of Pharmacy, Kariya Toyota General Hospital, 5-15 Sumiyoshi, Kariya City, Aichi 448-8505 Japan; 2 College of Pharmacy, Kinjo Gakuin University, 2- 1723 Omori Moriyama, Nagoya City, Aichi 463-8521 Japan; 3 Department of Pharmacy, Gifu Prefectural General Medical Center, 4-6-1, Noishiki, Gifu City, Gifu 500-8717 Japan; 4 Department of Urology, Kariya Toyota General Hospital, 5-15 Sumiyoshi, Kariya City, Aichi 448-8505 Japan

**Keywords:** Metastatic castration-resistant prostate cancer, Abiraterone, Prednisolone, Serum potassium level, Hypokalaemia

## Abstract

**Background:**

This study aimed to investigate the relationship between low-dose prednisolone (PSL) and the incidence of hypokalaemia at abiraterone acetate (abiraterone) plus PSL combination therapy targeting Japanese patients with metastatic castration-resistant prostate cancer (mCRPC).

**Methods:**

This retrospective observational study included 153 Japanese patients treated with abiraterone and PSL for mCRPC at Kariya Toyota General Hospital and Gifu General Medical Center between September 2014 and October 2022. The incidence of grade ≥ 2 hypokalaemia as well as serum potassium level variations and the continuous combination therapy duration were compared between the low-dose (5 mg/day of PSL) and the standard-dose (10 mg/day of PSL) groups.

**Results:**

This study included 153 patient of which 95 were matched to establish the analysis population. The low-dose and the standard-dose groups consisted of 13 and 82 patients, respectively. No significant difference in the incidence of grade ≥ 2 hypokalaemia was observed between the two groups [15.4% (2/13 patients) in the low-dose group and 12.2% (10/82 patients) in the standard-dose group, *P* = 0.667]. The low-dose group exhibited a decrease in serum potassium levels from 4.63 on day − 7 − 0 to 4.16 mmol/L on day 84 ± 10 (*n* = 7, *P* = 0.066), and serum potassium levels from day − 7 − 0 to 84 ± 10 in the low-dose group appeared to be great in the standard-dose group (*n* = 37, *P* = 0.475). The Kaplan–Meier curves for continuity of abiraterone and PSL therapy were not significantly different between the low-dose group (*n* = 13) and standard-dose group (*n* = 82, *P* = 0.427).

**Conclusion:**

Combination therapy with abiraterone and 5 mg/day of PSL in Japanese patients with mCRPC did not change the incidence of grade ≥ 2 hypokalaemia. However, although not significant, 5 mg/day of PSL demonstrated a decreasing trend in serum potassium levels with a larger degree of change than that of 10 mg/day of PSL. Therefore, the combination of abiraterone and 5 mg/day PSL can be administered to Japanese patients with mCRPC. The patients must be monitored for hypokalaemia through measurement of serum potassium levels and observation of subjective symptoms such as muscle weakness, convulsion etc. In addition, the doctor or the pharmacist must explain these symptoms to the patient and instruct them to consult their medical staff immediately in the event of development of such symptoms.

## Background

Abiraterone acetate (abiraterone), a selective inhibitor of cytochrome P450 17α-hydroxylase (CYP17A1), has been shown to suppress androgen synthesis in adrenal, testicular and prostate tumours [1, 2]. Phase 3 randomised control trials have revealed that among patients with metastatic castration-resistant prostate cancer (mCRPC), those in the abiraterone plus prednisone group had a longer overall survival than those in the placebo plus prednisone group (14.8 vs. 10.9 months, respectively) [3]. Other previous reports have shown that adding prednisone to abiraterone increased the treatment efficacy of mCRPC [1, 4–6]. The National Comprehensive Cancer Network Clinical Practice Guidelines recognises abiraterone as a novel hormone therapy for mCRPC [7]. Additionally, abiraterone plus prednisone is currently being used earlier during the treatment paradigm for prostate cancer to increase treatment exposure, which provides more benefits to the patients [1]. Japanese guidelines have also recommended abiraterone plus prednisolone (PSL) combination therapy as a standard primary treatment for mCRPC.

CYP17A1 inhibition by abiraterone, which significantly suppresses androgen and cortisol synthesis, has been shown to increase adrenocorticotropic hormone, which raises mineralocorticoid levels [1, 8, 9]. Hypokalaemia and urinal metabolite disorders have been identified as symptoms of apparent mineralocorticoid excess [1, 4, 5]. Therefore, adjustment of glucocorticoid dosage is required to prevent side effects caused by abiraterone. When using glucocorticoids for this purpose, 5 mg of PSL twice daily (10 mg/day) is recommended [5, 6, 10]. However, excessive exposure to glucocorticoids increases the risk for infections, adrenal insufficiency and decreased bone density [1, 11]. Given that abiraterone plus glucocorticoid combination therapy can be used at a relatively early phase of mCRPC, long-term use of this therapy can particularly cause excessive exposure to glucocorticoids [1].

Previous reports have shown that a dosage of < 10 mg/day of PSL plus abiraterone combination therapy can be effective for prostate cancer patients with high-risk prognostic factors who fail to respond to endocrine therapy [2, 12–15]. However, a randomised, open-label phase 2 study conducted in five countries reported that a lower prednisone dose appeared to mitigate the risk of hypokalaemia [1]. Subgroup analysis of the LATITUDE study targeting Japanese patients (i.e. comparison analysis of abiraterone plus 5 mg/day of PSL and placebo) reported that the abiraterone plus 5 mg/day of PSL group a lower death rate but higher incidence of grade 3/4 adverse events than did the placebo group [2]. However, no report targeting Japanese patients has investigated whether a dosage of < 10 mg/day of PSL plus abiraterone combination therapy would offer better safety over 10 mg/day of PSL plus abiraterone combination therapy, which is the standard treatment used in Japan. The current study investigated the relationship between low-dose PSL and the incidence of hypokalaemia at abiraterone plus PSL combination therapy targeting Japanese patients with mCRPC.

## Methods

### Study design and subjects

This retrospective observational study analysed the medical records of 153 Japanese patients who had previously received abiraterone and glucocorticoid combination therapy for mCRPC at Kariya Toyota General Hospital and Gifu General Medical Center between September 2014 and October 2022. The following cases were excluded: those with prostate cancer who had high-risk prognostic factors and failed to respond to endocrine therapy; those with missing data; those who self-interrupted administration, those who transferred to another hospital; those who received < 1,000 mg/day of abiraterone; and those who received dexamethasone instead of PSL. Furthermore, PSL dosage was excluded other than 5 mg and 10 mg. The included patients were divided into two groups according to the dosage of PSL (i.e. a low-dose group [5 mg/day of PSL] and a standard-dose group [10 mg/day of PSL]).

The primary endpoint includes the comparison of the higher incidence of grade ≥ 2 hypokalaemia between the low-dose and the standard-dose groups, and the secondary endpoint involves the amount of change of potassium between before and after combination therapy with abiraterone and either the low-dose or the standard-dose PSL and the continuity of combination therapy.

### Data collection

At the start of treatment, the following variables were collected: age; body surface area (BSA); Gleason score; metastases to the lymph nodes; bone and internal organs; cancer stage; detailed treatment history (number of chemotherapy lines after castration resistance, radiotherapy history and prostate surgery history); use of any medication that might increase serum potassium levels (e.g. potassium-sparing diuretic, angiotensin-converting enzyme inhibitor, angiotensin II receptor blocker or potassium supplements) or decrease them (e.g. loop diuretic, thiazide diuretic, calcium channel blockers or laxatives) and use of glucocorticoid before abiraterone initiation. Data on the following variables were extracted during the treatment period: duration of abiraterone administration, blood test findings (serum potassium [mmol/L], total bilirubin [T-Bil; mg/dL], aspartate transaminase [AST; U/L], alanine transaminase [ALT; U/L], blood urea nitrogen [BUN; mg/dL], estimated glomerular filtration rate [eGFR; mL/min/1.73m^2^], creatinine clearance [CCr; mL/min], prostate-specific antigen (PSA) [ng/mL] and adverse events (hypokalaemia) from initiating point of abiraterone (day − 7–0) to completing the administration or the end of investigation period.

### Hypokalaemia

The incidence of hypokalaemia was collected. Data on hypokalaemia of the highest grade were extracted during the treatment period. Hypokalaemia were determined according to the Common Terminology Criteria for Adverse Events version 5.0. The incidence of hypokalaemia was compared between the low-dose (5 mg/day of PSL) and the standard-dose (10 mg/day of PSL) groups. Serum potassium levels were analysed using blood tests conducted during the initiating point of abiraterone (day − 7–0) and day 84 ± 10 after initiation, which was the median duration of abiraterone and PSL combination therapy until the onset of grade ≥ 2 hypokalaemia.

### Continuity of abiraterone and PSL therapy

Continuity of abiraterone and PSL therapy was analysed using the duration of abiraterone administration in both groups.

### Statistical analysis

Normally distributed variables were expressed as mean ± standard deviation, whereas non-normally distributed variables were expressed as median (interquartile ranges). Unpaired parametric or nonparametric two-group comparisons were performed using the unpaired t-test or Mann–Whitney U test, respectively. Corresponding parametric or nonparametric two-group comparisons were performed using the paired t-test or Wilcoxon’s signed-rank test. Proportion comparisons were made using Fisher’s exact test. The Cochran–Mantel–Haenszel test for proportions was used for comparing the incidence of grade ≥ 2 hypokalaemia between the low-dose and the standard-dose groups. The stratification factor was based on the incidence of grade ≥ 2 hypokalaemia (yes vs. no) and use of the medication that might either increase or decrease serum potassium levels (yes vs. no). Time to onset of grade ≥ 2 hypokalaemia and continuity of abiraterone and PSL therapy were presented as Kaplan–Meier curves, with comparisons between the two groups being performed using the Log-rank test. All statistical analyses were conducted using SPSS version 24.0. The significance level was set at a risk rate of less than 5%.

## Results

### Patient characteristics

**Fig. 1:**
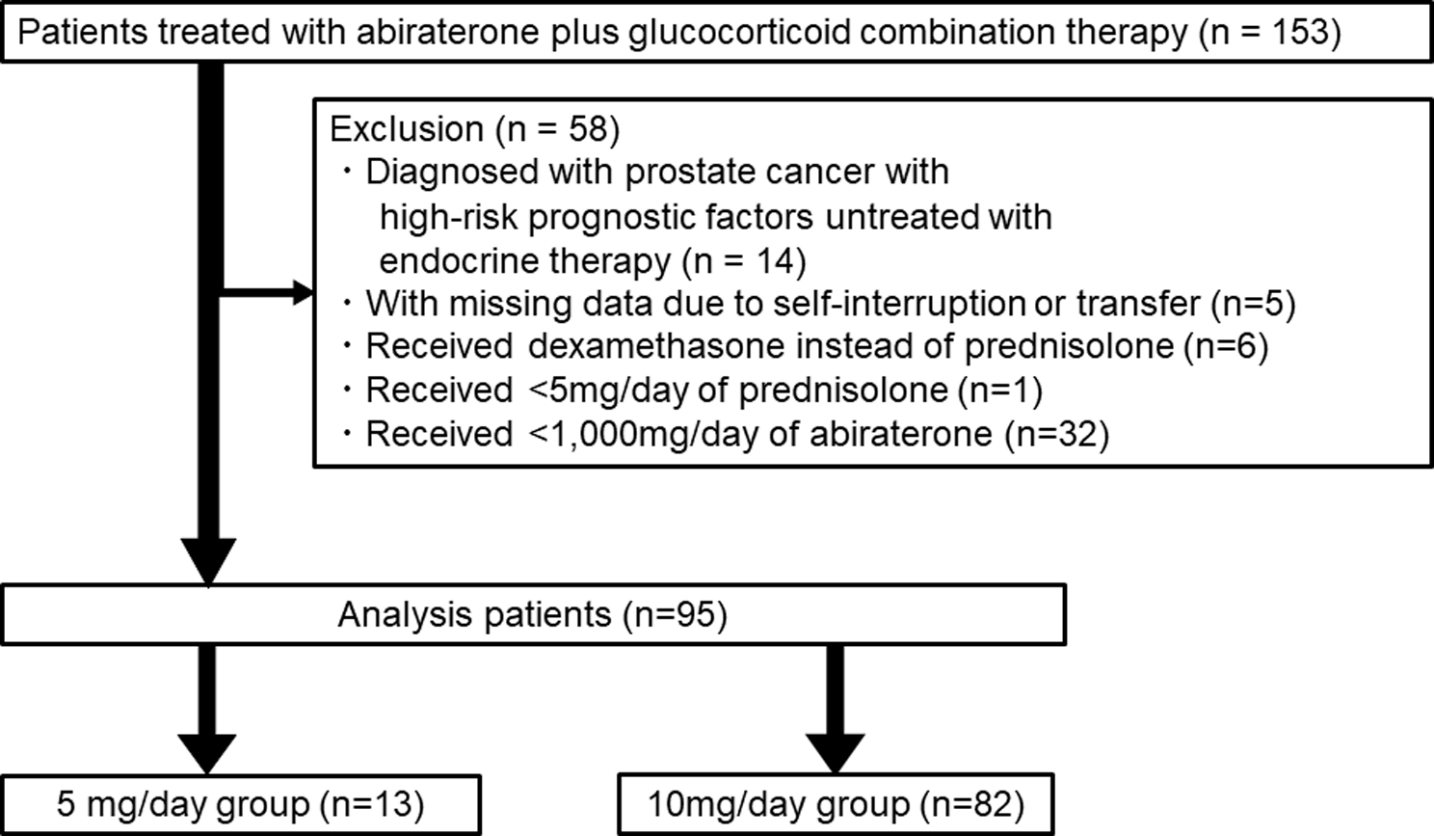
Classification of patients

This study included 153 patients who received abiraterone plus glucocorticoid combination therapy for mCRPC of which 95 were matched to establish the analysis population. Accordingly, the low-dose and the standard-dose group consisted of 13 and 82 patients, respectively (Fig. 1). The patient background information is provided in Table 1. Notably, there were no significant differences between the low-dose and the standard-dose groups. In terms of the reasons for choosing low-dose, 13 patients were included: 4 with diabetes, 1 at the patient’s request and 1 with hypokalaemia and diabetes. There were seven patients whose reasons could not be identified.

**Table 1:** Patient characteristics

	Low-dose group (*n* = 13)	Standard-dose group (*n* = 82)	*P* value
Age (year)	73 ± 5	74 ± 7	0.408
BSA (m^2^)	1.60 (1.50–1.86)	1.63 (1.54–1.70)	0.319
Gleason score	8 (8–9) (*n* = 11)	9 (8–9) (*n* = 76)	0.892
Metastasis			
Lymph nodes	7 (53.8%)	39 (47.6%)	0.769
Bone	11 (84.6%)	64 (78.0%)	0.729
Internal organs	1 (7.7%)	22 (26.8%)	0.177
Cancer stage	4 (4–4)	4 (4–4) (*n* = 80)	0.310
Number of chemotherapy lines	2 (1–3)	2 (2–3)	0.219
Radiotherapy history	5 (38.5%)	36 (43.9%)	0.772
Prostate surgery history	1 (7.7%)	24 (29.3%)	0.173
Use of medication that might increase potassium levels			
Diuretic	0 (0%)	3 (3.7%)	1.000
Antihypertensive	3 (23.1%)	20 (24.4%)	1.000
Potassium	0 (0%)	1 (1.2%)	1.000
Use of medication that might decrease potassium levels			
Diuretic	1 (7.7%)	6 (7.3%)	1.000
Antihypertensive	3 (23.1%)	21 (25.6%)	1.000
Laxative	3 (23.1%)	11 (13.4%)	0.400
Use of glucocorticoids prior to abiraterone initiation	2 (15.4%)	19 (23.2%)	0.726
Potassium (mmol/L)	4.4 (4.0–4.6)	4.2 (4.0–4.5)	0.242
T-Bil (mg/dL)	0.5 (0.4–0.6)	0.6 (0.4–0.7)	0.304
AST (U/L)	20 (16–23)	20 (17–26)	0.432
ALT (U/L)	17 (11–21)	15 (10–19)	0.389
BUN (mg/dL)	19.0 (16.0–20.0)	17.1 (14.8–20.8)	0.329
eGFR(mL/min/1.73 m²)	63.1 ± 17.0	70.9 ± 21.2	0.212
CCr (mL/min)	57.9 (53.5–74.8)	68.3 (50.0–83.7)	0.442
PSA (ng/mL)	25.9 (13.3–96.8)	12.7 (6.2–46.5)	0.229

BSA, body surface area; T-Bil, total bilirubin; AST, aspartate transaminase; ALT, alanine transaminase; BUN, blood urea nitrogen; eGFR, estimated glomerular filtration rate; CCr, creatinine clearance; PSA, prostate-specific antigen

Patient number and percentage (%) for the 13 cases in the low-dose (5 mg/day) group and 82 cases in the standard-dose (10 mg/day) group. Values for age and clinical examination are presented as mean ± standard deviation or median (interquartile ranges), respectively

### Investigation period

The median investigation period was 118 (61–222) and 150 (67–429) days in the low-dose and standard-dose groups, respectively. No significant differences in the investigation period were observed between both groups (*P* = 0.562).

### Incidence of hypokalaemia and change in serum potassium level

The incidence of grade ≥ 1 hypokalaemia was 38.5% (5 patients) and 28.0% (23 patients) in the low-dose and the standard-dose group, receptively, with no significant difference between the two groups (*P* = 0.516). Moreover, no significant difference in the incidence of grade ≥ 2 hypokalaemia were observed between the two groups (low-dose group: 15.4% [2 patients] vs. standard-dose group: 12.2% [10 patients], *P* = 0.667). Each group had once case of grade 4 hypokalaemia and actual serum potassium levels changed from 5.2 to 2.4 mmol/L in the low-dose group on day 392 and from 3.2 to 2.4 mmol/L in the standard-dose group on day 14. PSL plus abiraterone combination therapy was discontinued after causing grade 4 hypokalaemia. On the other hand, the supplementation of potassium preparations for grade 1 or 2 hypokalemia was carried out for just two cases, i.e., both low-dose group and standard-dose group have each one case. The serum potassium levels were restored after dosage immediately on each case.

Table 2 summarises the results in serum potassium level changes. The mean serum potassium level at day − 7–0 in the standard-dose group was lower than in the low-dose group (*P* = 0.019). However, the low-dose group demonstrated a decrease in serum potassium levels from day − 7–0 to 84 ± 10 (*P* = 0.066), which is greater than in the standard-dose group (*P* = 0.056).

**Table 2:** Comparison of changes in serum potassium level

	(Day − 7–0)	P value (Day − 7–0)	Day 84 ± 10	P value (Day − 7–0 vs. 84 ± 10)	Decrement value	P value (Decrement)	
Low-dose group (n = 7)	4.63 ± 0.40	0.019	4.16 ± 0.41	0.066	0.47 ± 0.56	0.056	
Standard-dose group (n = 37)	4.29 ± 0.33		4.23 ± 0.51	0.475	0.06 ± 0.50		

Serum potassium levels (mmol/L) on day − 7–0 and day 84 ± 10 and decrement value (change from day − 7–0 to day 84 ± 10) are presented as mean ± standard deviation. P values (day − 7–0) and P value (decrement) were compared between the low-dose (5 mg/day) and the standard-dose (10 mg/day) groups. P values (day − 7–0 vs. day 84 ± 10) were obtained from paired t-tests comparing the two groups

### Comparison of the incidence of grade ≥ 2 hypokinaemia considering the concomitant use of drugs that might affect potassium levels

The odds ratio for the incidence of grade ≥ 2 hypokalaemia due to the use of medication that could increase serum potassium levels was 1.307 (0.254–6.723), and did not significantly differ between the two groups (*P* = 0.900), while the odds ratio for the incidence of grade ≥ 2 hypokalaemia due to the use of medications that could decrease serum potassium levels was 1.306 (0.250–6.822), and did not significantly differ between the two groups (*P* = 0.895).

### Kaplan–Meier curves representing the time to onset of grade ≥ 2 hypokalaemia

**Fig. 2:**
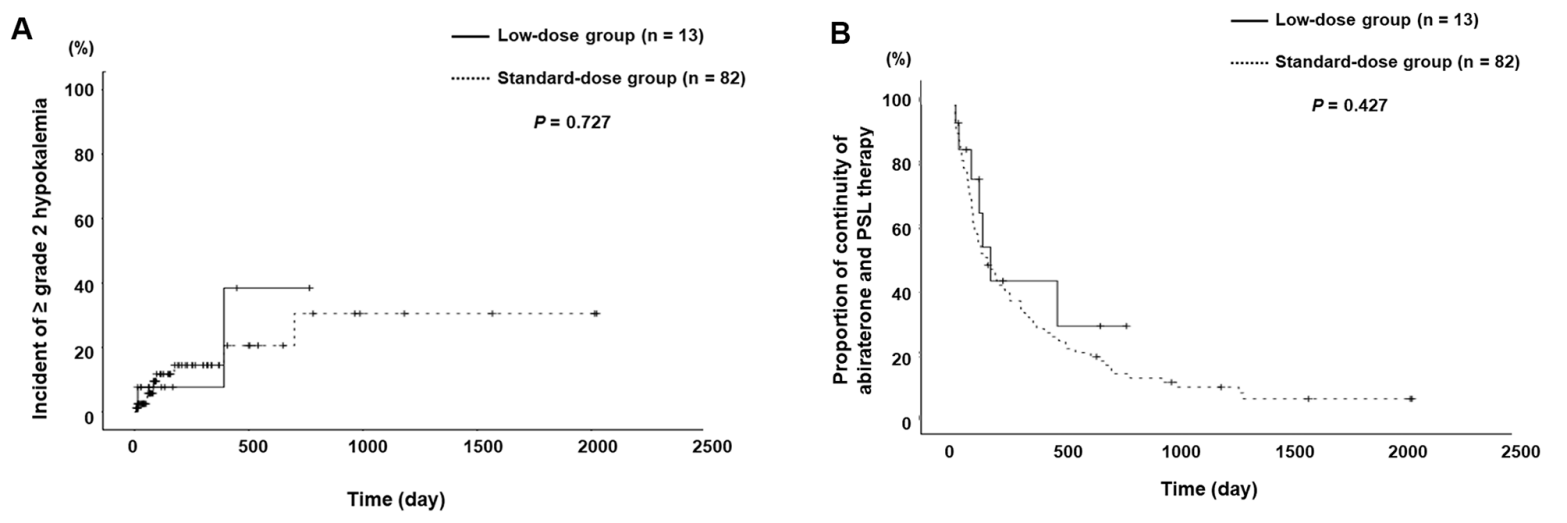
Kaplan–Meier curve. **A**) Incident of ≥ grade 2 hypokalaemia in the low-dose (5 mg/day) group (*n* = 13) and the standard-dose (10 mg/day) group (*n* = 82). **B**) Duration of combination therapy for the low-dose (5 mg/day) group (*n* = 13) and the standard-dose (10 mg/day) group (*n* = 82). PSL, prednisolone

The Kaplan–Meier curves representing the time to onset of grade ≥ 2 hypokalaemia showed no significant difference between the two groups (*P* = 0.727; Fig. 2A).

There was no significant difference in the duration of grade ≥ 2 hypokalaemia between the two groups (low-dose group: 118 [61–222] days vs. standard-dose group: 150 [67–429] days, *P* = 0.537).

### Kaplan–Meier curves for continuity of abiraterone and PSL therapy

The Kaplan–Meier curves for the continuation of abiraterone and PSL therapy showed no significant difference between the two groups (*P* = 0.427; Fig. 2B).

## Discussion

No significant difference in the incidence of hypokalaemia was observed between the low-dose and the standard-dose groups in both grade ≥ 2 hypokalaemia [15.4% (2/13 patients) and 12.2% (10/82 patients)] and grade ≥ 1 hypokalaemia [38.5% (5/13 patients) and 28.0% (23/82 patients)]. Extremely few grade 4 adverse events were reported in the current study, with each group exhibiting one case, which is consistent with some reports [3, 4]. The previous clinical trials in Japan revealed that the incidence of grade ≥ 1 hypokalaemia was 14.6% (7/48 patients) [10] and 8.5% (4/47 patients) [16] in 1000 mg/day of abiraterone with 10 mg/day of PSL. The current study revealed that the incidence of grade ≥ 1 hypokalaemia was higher than in these clinical trials. These trials may be conducted by strict criteria of patient selection, but we could not rule out the reason for the current study to demonstrate a high incidence of hypokalaemia. Although the incidence of grade ≥ 2 hypokalaemia was not affected due to the concomitant use of medications that could affect an increase or decrease in serum potassium levels in the current study, the other factors in the clinical background could have possibly affected the potassium levels. Thus, factor analysis is warranted in the future by increasing the sample size.

Bono et al. revealed that the incidence of hypokalaemia after administering 1000 mg/day of abiraterone and 10 mg/day of PSL was 17% (135/791 patients) for all grades [3]. Attard et al. demonstrated that the incidence of hypokalaemia of any grade after administering 1000 mg/day of abiraterone and various PSL dosages to 204 patients was 9.8% and 17.9–19.5% in those who received 10 and 5 mg/day of PSL, respectively [1]. The current study revealed a higher incidence of hypokalaemia for all grades in both the low-dose and standard-dose groups than previously reported. Taken together, Japanese patients with mCRPC may be susceptible to hypokalaemia. However, the current study could not completely conclude because no difference in the incidence of hypokalaemia was found between low-dose and 10 mg/day of PSL, as well as possible racial differences. We could not consider confounding factors because the incidence of grade ≥ 2 hypokalaemia as the primary endpoint was only reported in 10 and 2 patients in the standard-dose and low-dose groups, respectively. We plan to confirm the effect of hypokalaemia by PSL dosage with a larger sample size.

Considering the lack of studies on the changes in serum potassium level over time, the current study sought to determine the change in serum potassium levels from baseline to day 84 ± 10. Notably, low-dose group showed tendency to decrease in serum potassium level at day 84 ± 10 from baseline. However, a decrease in serum potassium level may be associated with changing aldosterone levels with low-dose PSL. This suggests the need for monitoring serum potassium levels during treatment. Moreover, serum potassium levels were not followed up at fixed points in all cases because of the retrospective study design. Increasing the number of cases or conducting a prospective clinical trial is necessary.

A wide variety of side effects have been reported for PSL. The degree and frequency of side effects are defined by the maximum dose per one dose of glucocorticoids and the cumulative dose over the entire period. Numerous studies have shown that 7.5–10 mg/day can increase the risk of osteoporosis, adrenal insufficiency, cardiovascular disease and glaucoma [17–21]. The risk of infection associated with steroids, including PSL, has been known to increase with increasing doses and longer durations of steroid use [22, 23]. However, recent studies have demonstrated that even at PSL doses that are as low as 5 mg/day, the risk of serious infections was 30% after 3 months, 46% after 6 months and 100% after 3 years from initiation, compared with that without the use of steroids; and the risk associated with the use of 5 mg/day of PSL for 3 years was determined to be equivalent to the use of 30 mg/day for a month [11, 24, 25]. To reduce the incidence of side effects other than hypokalaemia, the dosage of glucocorticoids must be minimized. A few cases were using < 5 mg/day of PSL in the current study. However, the dose-dependency of PSL of side effects other than hypokalaemia was not examined because of the small sample size, thereby prompting further investigation.

No significant difference in continuity of abiraterone and PSL therapy was observed between the low-dose and the standard-dose groups. This suggests that low-dose PSL did not interfere with the continuation of abiraterone plus glucocorticoid combination therapy, similar to 10 mg/day PSL. We further compared PSA levels between the low-dose and the standard-dose groups at initiation and treatment completion and found no difference between the two groups. However, we could not clearly determine the efficacy due to insufficient data.

Given that the current study was conducted based on information obtained from electronic medical records, the following limitations need to be considered: (1) some adverse events could have been overlooked and some information from patients could have been missed; (2) evaluating the safety factors of PSL and/or abiraterone, such as infection, primary bone fractures, elevated blood pressure [1], diarrhoea, nausea and vomiting was not possible given that this study was conducted mainly in an outpatient setting and the timing of data collection depended on the date of hospital visitation; (3) evidence indicating efficacy was insufficient. In light of these limitations, prospective clinical trials should be conducted to obtain more accurate data. Moreover, the current study included a small sample size. Also, the insufficient investigation of the following parameters was a limitation: (1) influence of patient background, (2) dose-dependency of PSL and (3) acquisition of more evidence regarding the influence of medication that might affect serum potassium levels.

Our results indicate that abiraterone plus 5 mg/day of PSL targeting Japanese patients with mCRPC is similar to 10 mg/day of PSL based on the incidence of grade ≥ 2 hypokalaemia. However, serum potassium levels are sufficiently monitored during combination therapy with abiraterone plus 5 mg/day of PSL. Additionally, prospective clinical trials are warranted to obtain more accurate data.

## Conclusion

Combination therapy with abiraterone plus 5 mg/day of PSL in Japanese patients with mCRPC is similar to 10 mg/day of PSL based on the incidence of grade ≥ 2 hypokalaemia. However, 5 mg/day of PSL demonstrated a decrease in serum potassium levels, which was greater than in 10 mg/day of PSL. Therefore, the combination of abiraterone and 5 mg/day PSL can be administered to Japanese patients with mCRPC. The patients must be monitored for hypokalaemia through measurement of serum potassium levels and observation of subjective symptoms such as muscle weakness, convulsion etc. In addition, the doctor or the pharmacist must explain these symptoms to the patient and instruct them to consult their medical staff immediately in the event of development of such symptoms.

## Data Availability

The datasets generated and analysed during the current study are not publicly available due the patients’ consent has not been obtained. but are available from the corresponding author on reasonable request.

## Abbreviations

Abiraterone: Abiraterone acetate
CYP17A1: Cytochrome P450 17α-hydroxylase
mCRPC: Metastatic castration-resistant prostate cancer
PSL: Prednisolone
BSA: Body surface area
T-Bil: Total bilirubin
AST: Aspartate transaminase
ALT: Alanine transaminase
BUN: Blood urea nitrogen
eGFR: Estimated glomerular filtration rate
CCr: Creatinine clearance
PSA: Prostate-specific antigen
